# Different toxic effects of YTX in tumor K-562 and lymphoblastoid cell lines

**DOI:** 10.3389/fphar.2015.00124

**Published:** 2015-06-17

**Authors:** Andrea Fernández-Araujo, Jon A. Sánchez, Amparo Alfonso, Mercedes R. Vieytes, Luis M. Botana

**Affiliations:** ^1^Department Farmacología, Facultad de Veterinaria, University Santiago de CompostelaLugo, Spain; ^2^Department Fisiología, Facultad de Veterinaria, University Santiago de CompostelaLugo, Spain

**Keywords:** Yessotoxin, apoptosis, autophagy, K-562, lymphoblastoid line

## Abstract

Yessotoxin (YTX) modulates cellular phosphodiesterases (PDEs). In this regard, opposite effects had been described in the tumor model K-562 cell line and fresh human lymphocytes in terms of cell viability, cyclic adenosine 3',5'-cyclic monophosphate (cAMP) production and protein expression after YTX treatment. Studies in depth of the pathways activated by YTX in K-562 cell line, have demonstrated the activation of two different cell death types, apoptosis, and autophagy after 24 and 48 h of treatment, respectively. Furthermore, the key role of type 4A PDE (PDE4A) in both pathways activated by YTX was demonstrated. Therefore, taking into account the differences between cellular lines and fresh cells, a study of cell death pathways activated by YTX in a non-tumor cell line with mitotic activity, was performed. The cellular model used was the lymphoblastoid cell line that represents a non-tumor model with normal apoptotic and mitotic machinery. In this context, cell viability and cell proliferation, expression of proteins involved in cell death activated by YTX and mitochondrial mass, were studied after the incubation with the toxin. Opposite to the tumor model, no cell death activation was observed in lymphoblastoid cell line in the presence of YTX. In this sense, variations in apoptosis hallmarks were not detected in the lymphoblastoid cell line after YTX incubation, whereas this type I of programmed cell death was observed in K-562 cells. On the other hand, autophagy cell death was triggered in this cellular line, while other autophagic process is suggested in lymphoblastoid cells. These YTX effects are related to PDE4A in both cellular lines. In addition, while cell death is triggered in K-562 cells after YTX treatment, in lymphoblastoid cells the toxin stops cellular proliferation. These results point to YTX as a specific toxic compound of tumor cells, since in the non-tumor lymphoblastoid cell line, no cell death hallmarks are observed.

## Introduction

Under unfavorable conditions, cells develop different strategies to survive and autophagy is the main mechanism; if survival is not possible, they activate autophagy cell death (Codogno and Meijer, 2005). Autophagy, also known as type II programmed cell death is activated after nutrient deprivation or with other stimulus, such as rapamycin treatment (Jung et al., 2010; Ouyang et al., 2012). In both cases, the target is the protein mTOR. This protein inhibits the autophagy through its active phosphorylated form (pmTOR). Under unfavorable conditions, the protein is dephosphorylated and inactivated, and as a result the autophagy cascade is triggered (Jung et al., 2010). Then, a complex network of metabolic pathways is activated to develop digestive vacuoles, called autophagosomes. These vacuoles are covered by type II of Light Chain 3 (LC3-II) protein (Codogno and Meijer, 2005; Stern et al., 2012). Three different human isoforms are described of LC3 protein: LC3A, LC3B, and LC3C (Wu et al., 2006). These types of LC3 protein are synthesized as the cytosolic type I LC3 protein (LC3A-I, LC3B-I, or LC3C-I) under normal conditions. When autophagy is activated, LC3-I is metabolized in type II (LC3A-II, LC3B-II, or LC3C-II) that binds to autophagosomes membrane (Lazova et al., 2012). Different parts of the cell and damaged organelles are digested by autophagosomes and fused with lysosomes (Klionsky and Emr, 2000). In this way, the basic elements to survive under unfavorable conditions are obtained (Cuervo et al., 2005). However, sometimes, autophagy degenerates in the digestion of the whole cell leading to the autophagy cell death (Tsujimoto and Shimizu, 2005). Another type of programmed cell death is apoptosis, also known as the type I of programmed cell death (Ouyang et al., 2012). Apoptosis can be activated through the intrinsic or mitochondrial apoptotic cell death or through the extrinsic or death receptor (DR) pathway (Elmore, 2007). The first one, known as mitochondrial apoptosis, is triggered by different stimulus that destabilizes mitochondrial membrane potential. As a result, the mitochondrial permeability transition pore (MPTP) is opened and pro-apoptotic proteins are released from the intermembrane space to the cytosol (Debatin et al., 2002). The pro-apoptotic proteins such as cytochrome c, smac/DIABLO, apoptotic inductor factor (AIF), and endonuclease G, activate first caspase 9 and then caspase 3 along with the rest of the apoptotic pathway (Javadov and Karmazyn, 2007). The extrinsic apoptotic cell death is triggered after the binding of a ligand to the DR that belongs to the tumor necrosis factor receptor family (TNFR), in the plasmatic membrane. Then, the death inducing signaling complex (DISC) is developed in the cytosolic region of the plasma membrane. This complex recruits and cleaves procaspases 8 and 10 into the active forms caspases 8 and 10. Caspase 8 also activates caspase 3 and converges at this point with the intrinsic apoptotic pathway (Korsnes and Espenes, 2011).

Yessotoxins (YTXs) are sulfated polyether compounds isolated for the first time from the scallops *Patinopecten yessoensis* (Murata et al., 1987). However, this group of toxins are synthesized by the dinoflagellates *Protoceratium reticulatum, Lingulodinium polyedrum, and Gonyaulax spinifera* (Satake et al., 1997; Paz et al., 2004; Rhodes et al., 2006). YTXs are modulators of phosphodiesterases (PDEs) and consequently affect the levels of cyclic adenosine 3′,5′-cyclic monophosphate (cAMP) (Alfonso et al., 2003, 2004, 2005; Pazos et al., 2006). The final effect is different depending on the cellular model studied, human fresh lymphocytes or human leukemic K-562 cell line (Alfonso et al., 2003; Tobío et al., 2012). Moreover, YTX has been described as a mitochondrial apoptosis inducer (Korsnes and Espenes, 2011; Korsnes, 2012). On the other hand, the structural protein A kinase anchoring protein 149 (AKAP149) binds PDE4A and protein kinase A (PKA) to the outer mitochondrial membrane (Asirvatham et al., 2004; Carlucci et al., 2008). These three components make a complex that is regulated by cAMP levels, since this second messenger activates PKA, and the whole complex moves around the cell depending on cAMP gradients (Baillie et al., 2005; Sample et al., 2012). Since YTX modulates PDEs, the complex was studied after toxin treatment in the tumor K-562 cell line. In this sense, a close relation between the complex expression and cell death activated by the toxin was discovered (Tobío et al., 2012; Fernandez-Araujo et al., 2014). This was supported by the fact that silencing the expression of PDE4A, the effect of YTX on K-562 cell viability is avoided and changes in the cytosolic expression of the rest of the proteins of the complex is observed (Fernandez-Araujo et al., 2014). In addition, a key role of PDE4A in apoptosis and autophagy cell death activated by YTX in the K-562 cell line has been observed (Fernández-Araujo et al., 2015). As mentioned, large differences, in terms of YTX toxicity, cAMP levels and AKAP149 expression, were found depending on the cellular model studied. In this sense, while no effect on cell viability was observed in human fresh lymphocytes, high cell death was detected in leukemic K-562 cells after YTX treatment (Tobío et al., 2012). Later on, the effect in the K-562 line was studied in depth and YTX was described as apoptotic and autophagy inductor in these cells (Fernandez-Araujo et al., 2014). As fresh lymphocytes have no mitotic capacity while leukemia cells are tumor cells, the aim of this work was to study the effect of YTX in a non-tumor cellular model with mitotic and apoptotic intact machinery in order to elucidate whether the toxic effects of YTX are exclusively for tumor cells or if they depend on the mitotic machinery. For this objective a non-tumor cell line, a lymphoblastoid cell line, was chosen. This cell line is a result of human B lymphocytes immortalized with the Epstein Barr virus, hence without tumor features (Sugimoto et al., 2004; Sie et al., 2009; Hussain and Mulherkar, 2012).

## Materials and methods

### Reagents and solutions

YTX was obtained from CIFGA Laboratories (Lugo, Spain). Anti-β-tubulin I, Bovine serum albumin (BSA), CaCl_2_, NaH_2_PO_4_, Trizma hydrochloride, Triton X-100, glycine, trizma base, SDS (sodium dodecyl sulfate) and Tween®20 were from Sigma-Aldrich (Madrid, Spain). NaCl, MgSO_4_, NaHCO_3_, and glucose were from Panreac (Barcelona, Spain). Anti-PDE4A and anti-LC3B was from ABCAM (CA, USA). Anti-Histone 1, anti-β-Actin, anti-pmTOR, anti-caspase 8 (active form), anti-cytochrome C, anti-rabbit IgG peroxidase conjugated, and Polyvinylidene fluoride (PVDF) membrane was from Millipore (Temecula, USA). Anti-Mouse IgG horseradish peroxidase-linked species-specific whole antibody was purchased from GE Healthcare (Barcelona, Spain).

Polyacrylamide gels and molecular weight marker Precision Plus Protein™ Standards Kaleidoscope™ were purchased from BioRad® (Barcelona, Spain). Protease Inhibitor Complete Tablets and Phosphatase Inhibitor Cocktail Tablets were from Roche (Spain). Free calcium and magnesium PBS used in flow cytometry assays was purchased from Gibco, Life Technologies (UK).

Physiological saline solution composition was (in mM): Na^+^ 142.3; K^+^ 5.94; Ca^2+^ 1; Mg^2+^ 1.2; Cl^−^ 126.2; HCO^−^_3_ 22.85; HPO^2−^_4_ 1.2, SO^2−^_4_ 1.2; glucose 1 g/L was added to the medium giving an osmotic pressure of 290 ± 10 mOsm/kg of H_2_O and pH was adjusted to 7.2 with HCl 0.1 N and CO_2_. PBS used to wash the western blotting membranes consisted of NaCl 137 mM; Na_2_HPO_4_ 10.14 mM; KH_2_PO_4_ 1.76 mM; KCl 2.68 mM; pH was adjusted to 7.2 with NaOH.

### Cell culture

K-562 cell line was purchased from the National Cancer Institute (NCI's, USA) and maintained in the Roswell Park Memorial Institute 1640 (RPMI 1640) medium supplemented with 10% fetal bovine serum (FBS) and 50 units/ml penicillin and 50 μg/ml streptomycin. All these reagents were from Gibco, Invitrogen (Spain). Cells were growing at 37°C in a humidified 5% CO_2_ atmosphere. Incubations with 30 nM YTX were carried out under these conditions of temperature, humidity and percentage of CO_2_.

Lymphoblastoid cell line was obtained from the Banco Nacional de ADN Carlos III (Spain). This cell line is a result of human B lymphocytes immortalized with the Epstein Barr virus, without tumor features and can be used for genetic or functional studies since these cells preserve the genetic characteristics of the lymphocyte B donor (Sugimoto et al., 2004; Sie et al., 2009; Hussain and Mulherkar, 2012). Lymphoblastoid cell line is maintained in the Roswell Park Memorial Institute 1640 (RPMI 1640) medium with HEPES and glutamine (from Biowest, France), supplemented with 15% fetal bovine serum (FBS), 100 units/ml penicillin and 100 μg/ml streptomycin. All these reagents were from Gibco (Invitrogen, Spain). Cells were growing at 37°C in a humidified 5% CO_2_ atmosphere. Incubations with 30 nM YTX were carried out under these conditions of temperature, humidity, and percentage of CO_2_.

### Subcellular fractionation

3 × 10^6^ cells per condition were incubated for 24 and 48 h with and without 30 nM YTX and then centrifuged and washed with saline solution. Cells were resuspended in lysis buffer with the following composition: 50 mM Tris-HCl, 150 mM NaCl, 1 mM EDTA, 1% Triton X-100, 1X Complete Protease Inhibitor and 1X Phosphatase Inhibitor Cocktail. The extract was sonicated and centrifuged (9300 g, 10 min, 4°C). The supernatant with the cytosolic fraction was transferred to a new tube and stored at −20°C for protein quantification.

### Western blotting analysis

Direct Detect Spectrometer from Millipore (Germany) was used to know sample protein concentration and BSA was used as protein standard. The electrophoresis run conditions were 200 V for 35 min. The cytosolic fraction was blotted to PVDF membrane with reduced SDS-PAGE. To determine the protein size and also to monitor the progress of the electrophoretic runs, a Precision Plus ProteinTM Standards Kaleidoscope™ molecular weight marker was used. After blockage with 0.5% BSA the membranes were incubated for 10 min with anti-cytochrome C, anti-PDE4A, anti-caspase 8, and anti-pmTOR, then were washed three times with PBS and 0.1% Tween®20 and incubated for 10 min with secondary anti-mouse or anti-rabbit IgG horseradish peroxidase-linked species-specific whole antibody. After three washes, chemiluminescence was visualized with SuperSignal® West Pico (low intensity) or with SuperSignal® West Femto (high intensity) both from Pierce (ThermoScientific, USA) and it was also used a Clarity Western ECL substrate (medium intensity) from BioRad®. The chemiluminiscent signal was detected with the Diversity GeneSnap software and analyzed by the Diversity 4 gel documentation and analysis system. Relative protein expression was calculated in relation to β-actin expression for each experiment in the cytosolic fraction. Experiments were carried out at least three times by duplicate. The purity of the subcellular fraction was tested by measuring control proteins from the cytosol fraction: Histone 1 was the negative control, as it is only present in the nuclear fraction and β-tubulin was the positive control because it is located in the cytosolic fraction.

### Cell viability

After the treatment with the toxin, 5 × 10^5^ cells per condition, cells were centrifuged (250 × g, 4 min, 4°C) and pellet and supernatant were separated. The pellets were first washed with saline solution and centrifuged (1100 × g, 5 min, 4°C), and then re-suspended in saline solution with MTT (250 μg/mL) (M2128, Sigma) and incubated for cell viability (Tobío et al., 2012). The supernatants were utilized to measure LDH release by using the *in vitro* Toxicology Assay Kit (TOX7, Sigma) following the commercial protocol.

### Mitochondrial mass

After the treatment with the toxin, cells were washed with saline solution, centrifuged (1200 × g, 5 min, 4°C) and incubated with 200 nM MitoTracker® Deep Reed FM (Invitrogen, USA) for 30 min, at 37°C in dark. Then the cells were washed and fixed with 4% paraformaldehyde (PFA) for 20 min at 4°C. Next, the cells were washed twice with PBS free of calcium and magnesium and finally resuspended in 80 μL PBS for flow cytometry. The data were analyzed with the IDEAS 4.0 Cell Image Analysis software. The assays were performed with 1 × 10^6^ cells per condition.

### Cell proliferation

Cell proliferation was measured by using three methods, the Scepter™ Handheld automated cell counter (Millipore, Spain) and two methods for total cellular protein determination, Bradford (Bio-Rad protein Assay) and the Direct Detect™ Spectrometer (Millipore, Germany). The number of cells was corrected in each experiment with the cell viability data by using the MTT assay. After the treatment with 30 nM of YTX for 24 and 48 h, 6 × 10^5^ cells per condition, cells were centrifuged (600 × g, 5 min, 4°C), and supernatants were eliminated. The pellets were first washed with saline solution and centrifuged (600 × g, 5 min, 4°C), and then re-suspended in saline solution 250 μL. Hundred microliter of cellular suspension were used to count the number of cells with the Scepter™ Handheld automated cell counter. One hundred and fifty microliter were centrifuged, diluted in 50 μl of water and sonicated. Then, the concentration of protein was determined both, by Bradford assay and Direct Detect™ (Millipore, Spain). A calibration curve of number of cells (750,000 from 50,000) vs. concentration of protein was done in each experiment and the linearity was always higher than 0.99 for both cellular lines.

### Statistical analysis

All the experiments were carried out at least three times by duplicate. ANOVA was used to examine statistical significance, assumed for *p* < 0.05. Results were expressed as the means ± SEM.

## Results

**Figure 1:**
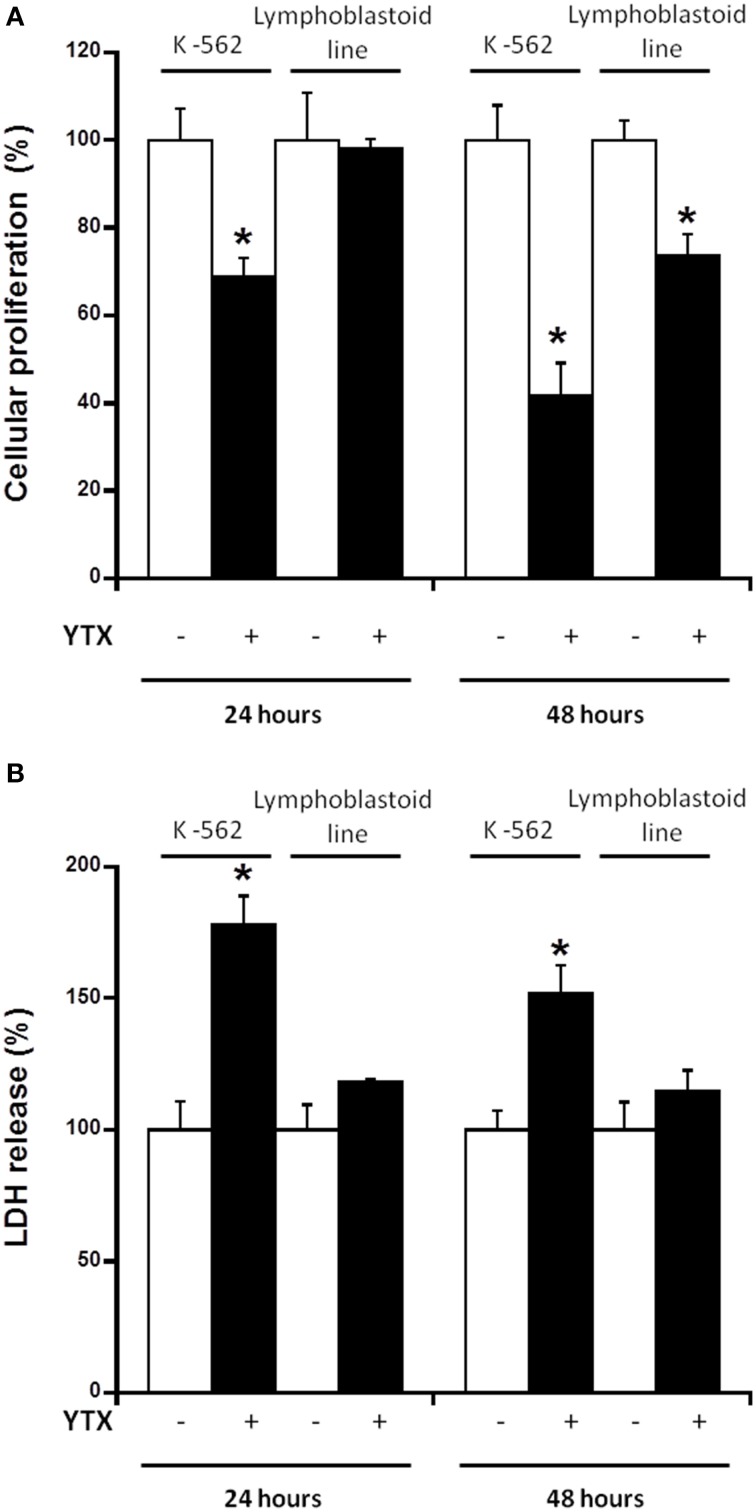
**Effect of YTX on cellular MTT signal and LDH release in K-562 and lymphoblastoid cell lines**. Cells were incubated with 30 nM YTX for 24 and 48 h (37°C and 5% CO_2_ atmosphere). **(A)** Percentage of cellular proliferation measured by MTT assay in K-562 and lymphoblastoid cell lines after 24 and 48 h of YTX incubation. **(B)** Percentage of LDH release of K-562 and lymphoblastoid cell lines after 24 and 48 h of YTX incubation. Mean ± SEM of three experiments. ^*^Significant differences between untreated and YTX-treated cells in each case by ANOVA test.

Opposite effects of YTX were reported in tumor K-562 cell line and fresh human lymphocytes (Tobío et al., 2012). This may be due to the differences between these cellular models, since fresh human lymphocytes do not have the ability to grow by themselves. Therefore, it would be interesting to compare the effect of YTX in two cellular lines with similar mitotic machinery. For this purpose, the lymphoblastoid cell line was chosen as a non-tumor model, and compared with the K-562 tumor cell line model. The lymphoblastoid cell line can grow and has normal apoptotic machinery, while the K-562 cell line has not (Hussain and Mulherkar, 2012). Figure 1 shows cellular proliferation, measured by MTT metabolization, and plasma membrane integrity measured by LDH release (Figures 1A,B, respectively) in both cell lines after 24 and 48 h of toxin exposure. A 32% decrease in cell viability was observed in K-562 cell line after 24 h with YTX. However, no effect was observed in lymphoblastoid line under the same conditions. After 48 h of treatment, K-562 cell viability was decreased by 59% while the reduction in lymphoblastoid cell line was 27%. On the other hand, after 24 h of YTX treatment, LDH release was 78% increased in the K-562 cell line, while at this time YTX did not induce any effect in LDH release in lymphoblastoid cells. After 48 h in the presence of YTX, the increase in LDH release was 52% in K-562 cells, while no effects were observed in lymphoblastoid cells.

**Figure 2:**
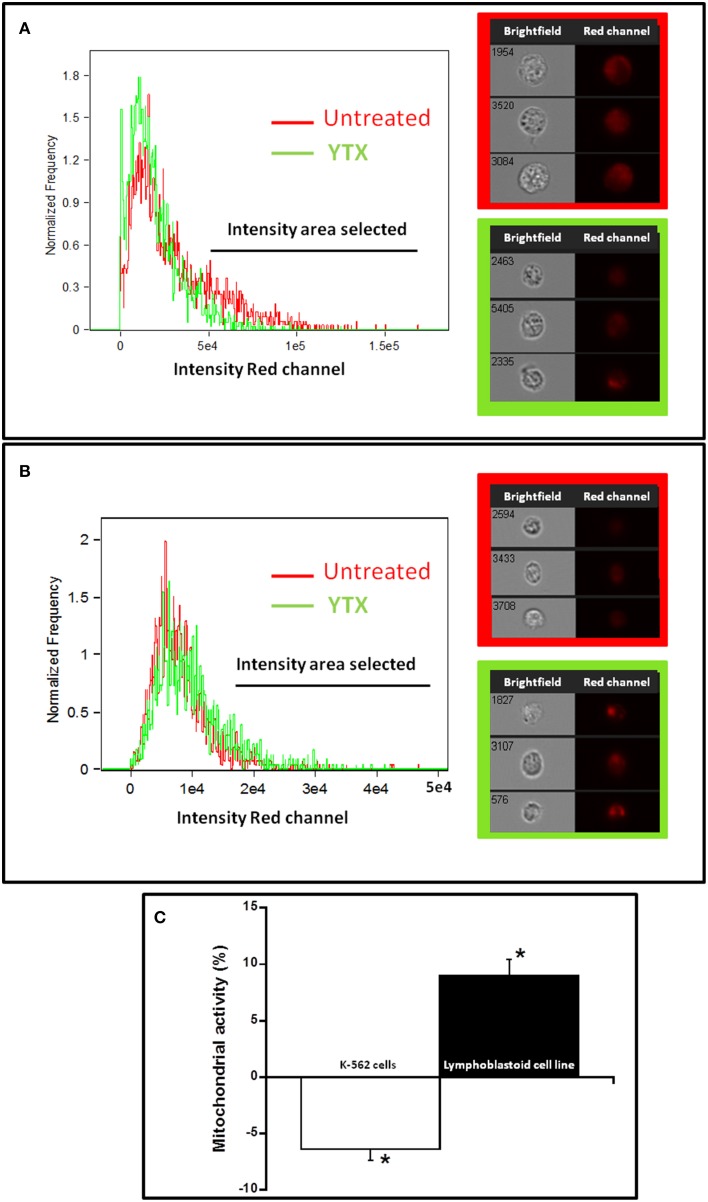
**Effect of YTX on total mitochondrial mass of K-562 and lymphoblastoid cell lines**. Representative cellular images and histograms of the mitochondrial mass intensities in K-562 and lymphoblastoid cell lines after 48 h (**A,B**, respectively) of YTX incubation (37°C and 5% CO_2_ atmosphere). Brightfield images and red channel images with MitoTracker®Deep Reed FM intensity are represented to each cell model on the right of the histograms. **(C)** Percentage of cells with mitochondrial mass intensities selected of K-562 and lymphoblastoid cell lines after 48 h of YTX treatment. Data referred to untreated cells. Mean ± SEM of three experiments (5000 cells were analyzed in each experiment). ^*^Significant differences between YTX-treated and untreated cells by ANOVA test.

The result obtained after 48 h of YTX treatment in lymphoblastoid cells with MTT, show a decrease in cellular proliferation but not in LDH release. Since the MTT dye is metabolized by the mitochondria, the fall of MTT signal observed may be caused either by a decrease in mitochondrial mass or by a decrease in the cell number. To find out which of the two options was going on, a dye to measure mitochondrial mass was used and in this way to know the direct effect of YTX in this organelle by using imaging flow cytometry. Therefore, in Figure 2 mitochondrial mass is shown both in K-562 cell line and in lymphoblastoid line. When the MitoTracker®Deep Reed FM dye is metabolized by the mitochondria, the intensity in red channel is increased, therefore this signal is proportional to mitochondria quantity. Figures 2A,B represent mitochondrial mass of K-562 cells and lymphoblastoid cells, respectively, after 48 h of YTX incubation. Higher X-axis values represent more dye intensity, hence more quantity of the mitochondria. Figure 2C represents the mean of three experiments. This graphic shows a significant 6% decrease of mitochondrial intensity after 48 h of incubation with YTX in a K-562 cell line, while the intensity is 9% increased in the lymphoblastoid line. Therefore, the decrease in MTT signal obtained in Figure 1A with the lymphoblastoid line after 48 h of treatment with YTX is probably not due to a decrease in mitochondrial mass but probably due to a decrease in the cell number with respect to the control without treatment.

**Figure 3:**
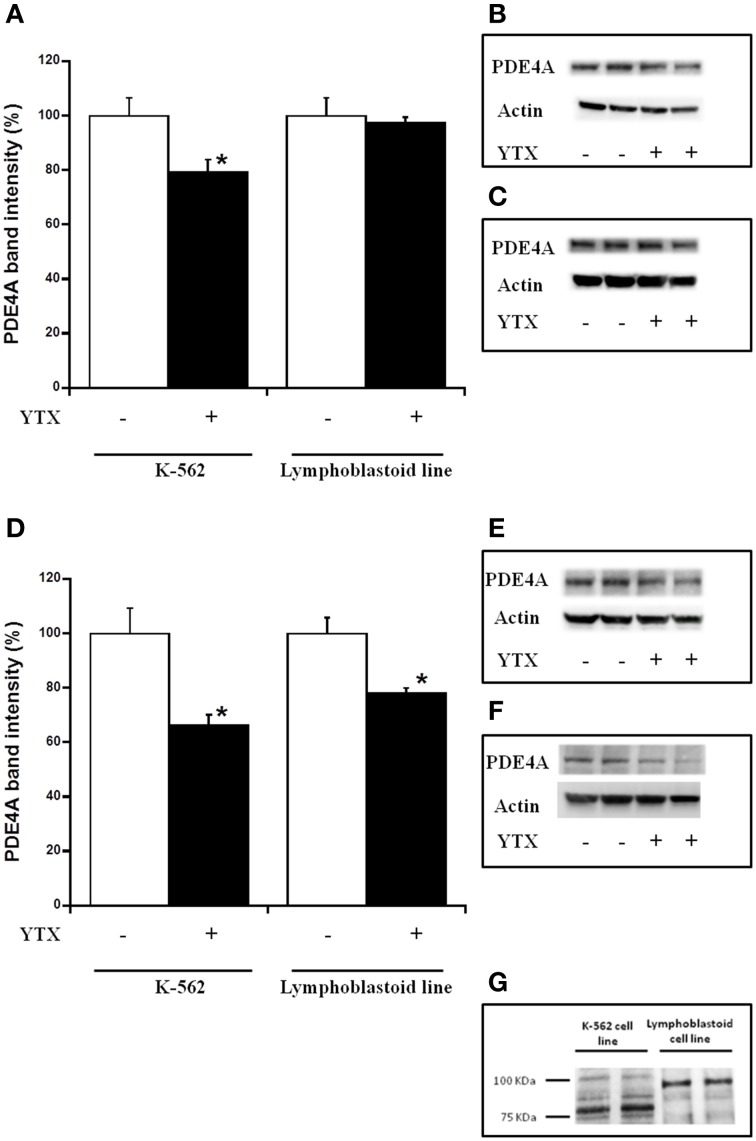
**Effect of YTX on cytosolic PDE4A expression in K-562 and lymphoblastoid cell**. Cells were incubated for 24 and 48 h with 30 nM YTX (37°C and 5% CO_2_ atmosphere). **(A,D)** Percentage of cytosolic PDE4A levels in K-562 and lymphoblastoid cell lines after 24 and 48 h of YTX incubation, respectively. Mean ± SEM of three experiments. Cytosolic PDE4A values were calculated respect to β-actin band intensity. 3 × 10^6^ cells per condition were lysed and 20 μg of total protein per condition was charged in the electrophoresis gel. ^*^Significant differences between untreated and YTX-treated cells by ANOVA test. **(B,C)** Representative experiments of western blot band intensity of cytosolic PDE4A in K-562 (~80 KDa) and lymphoblastoid (~98 KDa) cell lines after 24 h of treatment, respectively. **(E,F)** Representative experiments of western blot band intensity of cytosolic PDE4A in K-562 (~80 KDa) and lymphoblastoid (~98 KDa) cell lines after 48 h of treatment, respectively. **(G)** PDE4A band of K-562 and lymphoblastoid cell lines in western blot membranes. PDE4A antibody targets to a PDE4A protein with a molecular weight of 98 KDa.

It has been described the key role of PDE4A in the mechanism of action of YTX (Fernandez-Araujo et al., 2014). Therefore, this protein was studied in the lymphoblastoid cell line and compared to the tumor K-562 cell line after YTX treatment. Figure 3 shows the PDE4A expression in both cell models after 24 and 48 h (Figures 3A,D, respectively) of exposure to the toxin. While a 25% decrease in PDE4A expression was observed in K-562 cells after 24 h of treatment, no variations were detected in the lymphoblastoid cell line (Figure 3A). However, as Figure 3D shows, a decrease of 34 and 22%, in PDE4A levels was observed in K-562 and lymphoblastoid lines, respectively, after 48 h of toxin treatment. Therefore, cytosolic PDE4A levels are also involved in YTX effect in lymphoblastoid cell line. Surprisingly, according to the western blot experiments, different molecular weights for PDE4A proteins were observed depending on cellular model use. Figure 3G shows the PDE4A band with a lower molecular weight, around 80 KDa, in the case of K-562 cells, and 98 KDa band in lymphoblastoid cells. This is the normal molecular weight that targets the anti-PDE4A used in these experiments.

**Figure 4:**
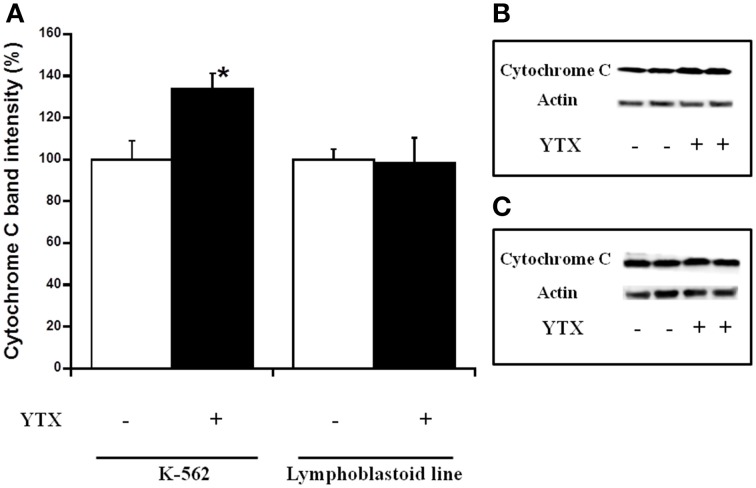
**Effect of YTX on cytosolic cytochrome c expression in K-562 and lymphoblastoid cell lines after 24 h of incubation**. Cells were incubated for 24 h with 30 nM YTX (37°C and 5% CO_2_ atmosphere). **(A)** Percentage of cytosolic cytochrome c levels in K-562 and lymphoblastoid cell lines after 24 h of YTX incubation. Mean ± SEM of three experiments. Cytosolic cytochrome c values were calculated respect to β-actin band intensity. 3 × 10^6^ cells per condition were lysed and 20 μg of total protein per condition was charged in the electrophoresis gel. ^*^Significant differences between untreated and YTX-treated cells by ANOVA test. **(B,C)** Representative experiments of western blot band intensity of cytosolic cytochrome c in K-562 and lymphoblastoid cell lines after 24 h of treatment, respectively.

**Figure 5:**
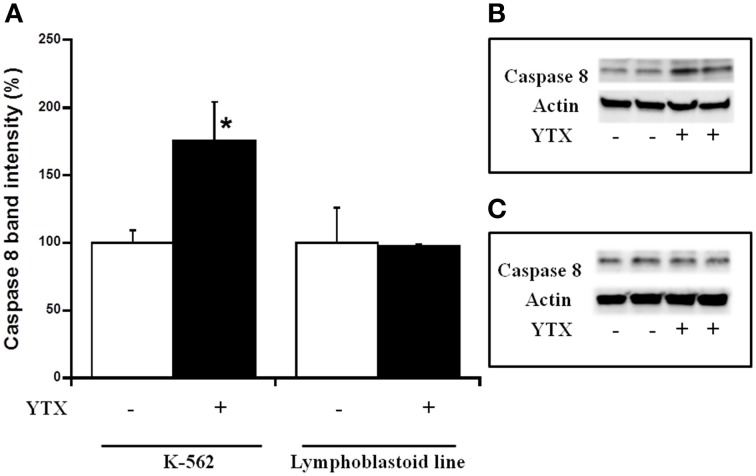
**Effect of YTX on cytosolic caspase 8 expression in K-562 and lymphoblastoid cell lines after 24 h of incubation**. Cells were incubated for 24 h with 30 nM YTX (37°C and 5% CO_2_ atmosphere). **(A)** Percentage of cytosolic caspase 8 levels in K-562 and lymphoblastoid cell lines after 24 h of YTX incubation. Mean ± SEM of three experiments. Cytosolic caspase 8 values were calculated respect to β-actin band intensity. 3 × 10^6^ cells per condition were lysed and 20 μg of total protein per condition was charged in the electrophoresis gel. ^*^Significant differences between untreated and YTX-treated cells by ANOVA test. **(B,C)** Representative experiments of western blot band intensity of cytosolic caspase 8 in K-562 and lymphoblastoid cell lines after 24 h of treatment, respectively.

**Figure 6:**
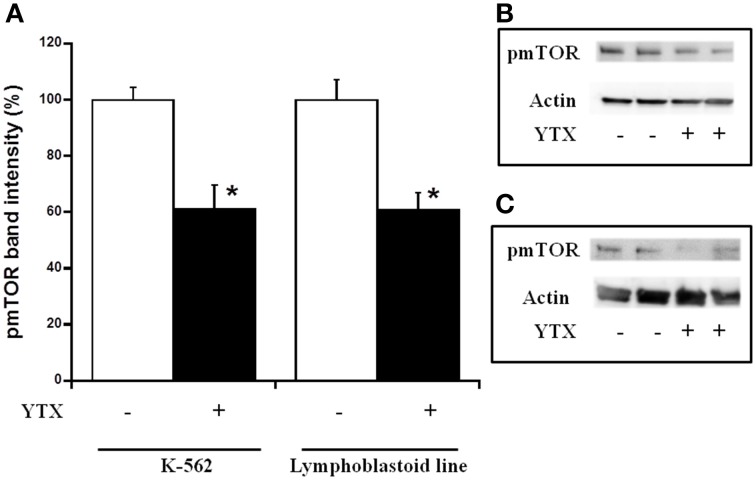
**Effect of YTX on cytosolic pmTOR expression in K-562 and lymphoblastoid cell lines after 48 h of incubation**. Cells were incubated for 48 h with 30 nM YTX (37°C and 5% CO_2_ atmosphere). **(A)** Percentage of cytosolic pmTOR levels in K-562 and lymphoblastoid cell lines after 48 h of YTX incubation. Mean ± SEM of three experiments. Cytosolic pmTOR values were calculated respect to β-actin band intensity. 3 × 10^6^ cells per condition were lysed and 20 μg of total protein per condition was charged in the electrophoresis gel. ^*^Significant differences between untreated and YTX-treated cells by ANOVA test. **(B,C)** Representative experiments of western blot band intensity of cytosolic pmTOR in K-562 and lymphoblastoid cell lines after 48 h of treatment, respectively.

**Figure 7:**
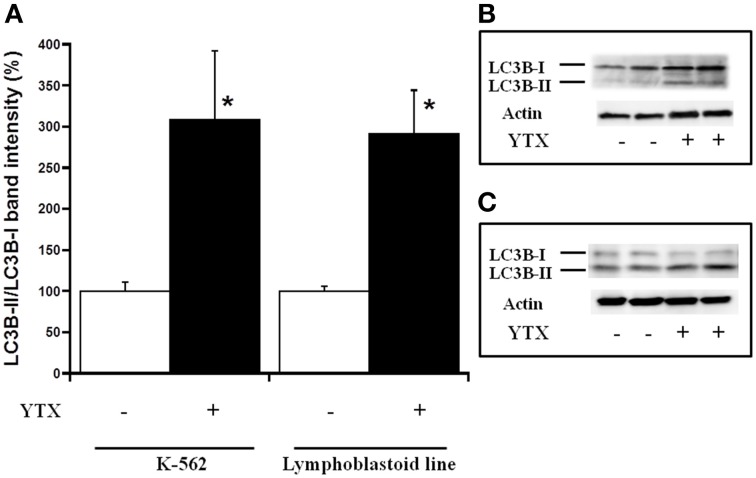
**Effect of YTX on cytosolic LC3B-II/LC3B-I expression in K-562 and lymphoblastoid cell lines after 48 h of incubation**. Cells were incubated for 48 h with 30 nM YTX (37°C and 5% CO_2_ atmosphere). **(A)** Percentage of cytosolic LC3B-II/LC3B-I levels in K-562 and lymphoblastoid cell lines after 48 h of YTX incubation. Mean ± SEM of three experiments. Cytosolic LC3B-I and LC3B-II values were calculated respect to β-actin band intensity and the ratio between LC3B-II and LC3B-I was calculated in order to quantify the autophagosomal LC3B-II isotype 3 × 10^6^ cells per condition were lysed and 20 μg of total protein per condition was charged in the electrophoresis gel. ^*^Significant differences between untreated and YTX-treated cells by ANOVA test. **(B,C)** Representative experiments of western blot band intensity of cytosolic LC3B-I and LC3B-II in K-562 and lymphoblastoid cell lines after 48 h of treatment, respectively.

**Figure 8:**
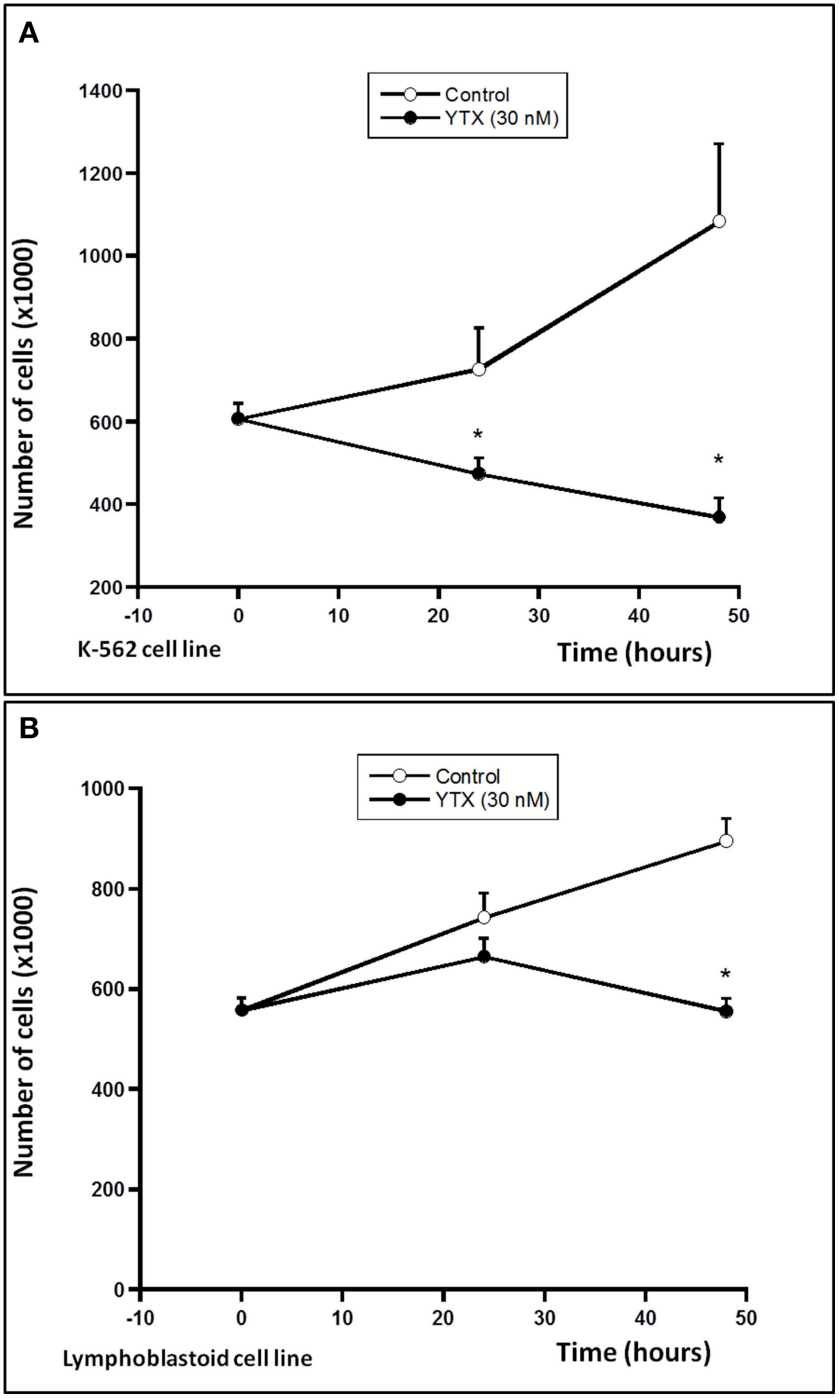
**Effect of YTX on cellular proliferation in K-562 and lymphoblastoid cell lines**. Cells were incubated with 30 nM YTX for 24 and 48 h (37°C and 5% CO_2_ atmosphere). Cells were counted with the Scepter™ Handheld automated cell counter and confirmed as final concentration of protein by Direct Detect™ and Bradford assay. **(A)** Number of K-562 cells after 24 and 48 h of YTX incubation. **(B)** Number of lymphoblastoid cells after 24 and 48 h of YTX incubation. Mean ± SEM of four experiments. ^*^Significant differences between untreated and YTX-treated cells in each case by ANOVA test.

Either, the activation of both intrinsic and extrinsic apoptosis processes through PDE4A modulation in K-562 cells after 24 h YTX incubation, as well as the autophagy cell death triggered through the PDE4A modulation in the same cell line after 48 h of YTX treatment were extensively studied (Fernandez-Araujo et al., 2014; Fernández-Araujo et al., 2015). Therefore, the activation of these pathways was checked in the lymphoblastoid cell line. First, different apoptotic hallmarks were studied under these conditions. Figure 4 shows the expression of the mitochondrial apoptotic marker cytochrome c. While in the K-562 cell line a 34% increase in cytosolic cytochrome c levels after 24 h YTX incubation, no effects were observed in lymphoblastoid cells. The same happens when the typical extrinsic apoptotic hallmark, the active form of caspase 8, was measured. As Figure 5 shows, caspase 8 levels were 75% increased in the tumor K-562 cell line in the presence of YTX for 24 h, while in these conditions no effect was observed in the lymphoblastoid line. Therefore, contrary to K-562 cell line, after 24 h of incubation in the presence of YTX apoptosis was not activated in the lymphoblastoid cell line. Since autophagy was trigged in the K-562 cell line after 48 h of YTX incubation, next the active form of mTOR (pmTOR), as one representative autophagy hallmark, was checked in the lymphoblastoid cell line after 48 h incubation with the toxin (Codogno and Meijer, 2005). Surprisingly, similar results were obtained in both K-562 and lymphoblastoid cell lines. A decrease of 39 and 40% was observed after 48 h of YTX treatment in the tumor and in the non-tumor cell line, respectively (Figure 6). Furthermore, Figure 7 shows the other autophagic hallmark studied in both cell lines, the LC3B-II/LC3B-I ratio. Again very similar results were obtained in both cellular models, since after 48 h of YTX incubation an increase of 208% in LC3B-II/LC3B-I ratio was observed in K-562 cells and 190% in lymphoblastoid cell line. Therefore, these results suggest the activation of an autophagic pathway in the lymphoblastoid cell line, but it does not imply the induction of cell death since no LDH release was observed as Figure 1B shows. However, to confirm this theory, the proliferation of both cell lines was studied, Figure 8. K-562 cells grow up from near to 6 × 10^5^ cells at the beginning of the experiment, time 0, to around 7.3 × 10^5^ after 24 h and to around of 11 × 10^5^ after 48 h (Figure 8A). However, after 24 h of YTX incubation, the number of cells has decreased to 4.7 × 10^5^ cells, and after 48 h of YTX treatment, the number of cells has fallen to near 3.7 × 10^5^ cells. These results mean a decrease in cells number after YTX incubation and significant differences were obtained in untreated and YTX-treated cells. On the other hand, lymphoblasoid cell line shows another pattern of proliferation (Figure 8B). The total number of cells in control population was around 5.6 × 10^5^, 7.4 × 10^5^, and 9 × 10^5^ at time 0, after 24 and 48 h, respectively. However, after 24 h of YTX treatment, the number of cells was near to 6.64 × 10^5^, and after 48 h of YTX incubation, the number of cells was around 5.5 × 10^5^. No significant differences in the number of cells were observed after 24 and 48 h of YTX treatment compared to time 0, therefore neither cell death nor growth was detected. Also, no differences were detected between untreated and YTX-treated cells after 24 h of YTX incubation. However, the number of cells after 48 h of YTX treatment was significantly lower compared to untreated cells at this time. Therefore, the proliferation of lymphoblastoid cell line was arrested by YTX effect.

## Discussion

YTX activates different cell death types depending on the cellular model (Korsnes, 2012). Moreover, opposite effects in terms of cell viability were obtained after the treatment with YTX of the tumor K-562 cell line and fresh human lymphocytes (Tobío et al., 2012). These observations point to YTX as an interesting compound to target tumor cell lines but not normal cells (Botana et al., 2011). However, there are several differences between an immortalized cellular line and fresh primary cells (Baserga, 1965). In this context, the comparative study of YTX effect in a tumor cell line and a non-tumor cell line could give interesting information about the mechanism of action of YTX. The pathways activated in the leukemic K-562 cell line by this toxin were widely studied. It was described that PDE4A shows a key role in YTX effect that leads to the activation of different programmed cell death types (Tobío et al., 2012; Fernandez-Araujo et al., 2014; Fernández-Araujo et al., 2015). The lymphoblastoid cell line used in this paper is a result of human B lymphocytes immortalized with the Epstein Barr virus, hence without tumor features and with intact apoptotic and mitotic machineries (Sugimoto et al., 2004; Sie et al., 2009; Hussain and Mulherkar, 2012). Therefore, this line is a good tool to study the effects of the toxin in a non-tumor cellular model with mitotic ability to grow up (fresh cells do not have this property), and normal apoptotic machinery (tumor cells do not have it) (Sugimoto et al., 2004; Sie et al., 2009; Hussain and Mulherkar, 2012).

The first difference observed between K-562 and lymphoblastoid lines was the effect of YTX on cell viability. Cellular proliferation is usually measured by MTT assay. This assay informs about the percentage of viable cells capable to reduce the MTT dye within the mitochondria (Mosmann, 1983; Loveland et al., 1992; Verma et al., 2010). The quantification of LDH release is a simple assay to know cell membrane integrity, since after the lysis of the cells, LDH is released to the extracellular medium and the released amount is proportional to the number of broken cells (Lobner, 2000). In this regard, the K-562 cell line did show a decrease in cell proliferation accompanied by an increase in LDH release after both 24 and 48 h of YTX incubation. That is, after YTX treatment the number of K-562 cells was decreasing because cell death was triggered, since apoptosis and autophagy activation was observed, the same as it was described in previous studies (Fernandez-Araujo et al., 2014; Fernández-Araujo et al., 2015). However, in the lymphoblastoid cell line, no effect in cell viability was observed in the first 24 h of treatment, and after 48 h, cell proliferation was decreased without any LDH release, that is without cellular lysis. These results suggest no cell death activation but probably proliferation arrested in the lymphoblastoid line after a long term of YTX exposure. The decrease in the signal observed in MTT assay in the lymphoblastoid line after 48 h YTX treatment comparing with cells without treatment is due to a non-increase in the cell number in the same way than the control where the cells can proliferate. In addition at this time, an increase in the mitochondrial mass, studied by flow cytometry, was observed. In this sense, while the lymphoblastoid cell line usually duplicate its population in 24 h (Sie et al., 2009), YTX could block this increase in the population without cell death activation.

Interesting, the increase in caspase 8 activity was previously observed in K-562 cell line after 24 h of YTX treatment (Fernandez-Araujo et al., 2014). In the present paper, the expression of the active form of caspase 8 is also observed in K-562 cells. This active hallmark is typical from extrinsic apoptotic pathway, and has never been discussed after YTX incubation (Korsnes and Espenes, 2011; Korsnes, 2012; Fernandez-Araujo et al., 2014). In addition, the levels of cytochrome c demonstrate that YTX is also triggering the intrinsic apoptosis (Fulda and Debatin, 2006). Therefore, YTX activates both intrinsic and extrinsic apoptotic cell death. However, none of these hallmarks were observed in lymphoblastoid cells, showing different YTX effects depending on the cellular model, as it was previously pointed out (Tobío et al., 2012).

PDE4A is involved in the mechanism of action of YTX in the K-562 cell line (Fernandez-Araujo et al., 2014). However, after 24 h of YTX treatment, the levels of cytosolic PDE4A were not modified in the lymphoblastoid cell line, and at this time apoptotic hallmarks and cell viability were not modified either. Moreover, when PDE4A was studied by western blot, different molecular weights for both K-562 cell line and lymphoblastoid cells were observed. This fact can be relevant since any difference in the structure of the PDE4A protein could be a key point of the mechanism of action of YTX. The molecular structure of proteins is a critical parameter for the affinity or interaction between proteins and other molecules, as it was reported in previous studies (Funder et al., 1974; Yatime et al., 2011; Tubaro et al., 2014). These differences can lead to different YTX-PDE4A affinity or to different PDE4A functions depending on the PDE4A structure. In this sense, tumor cells have protein mutations that could lead to the change in the protein function, and these mutations can be used as tumor biomarkers (Wang et al., 2011). Therefore, the PDE4A on the K-562 cell line seems to be the specific and maybe a potential target to tumor therapies in this type of leukemia.

After 48 h of YTX treatment, lymphoblastoid cells showed similar features to those observed in the K-562 cell line (cytosolic PDE4A and pmTOR decrease and LC3B-II/LC3B-I ratio increase). However, these effects did not imply cell death in the lymphoblastoid cell line, as LDH results have shown. At this time, 48 h of YTX treatment, the autophagy activated in K-562 leads to cell death. However, in the lymphoblastoid cell line, autophagy pathway is activated, as the pmTOR and LC3B-II/LC3B-I expression shows. Under unfavorable conditions cells develop mechanisms to start the digestion or recycling of different parts of the cell in order to obtain elements to survive under these critical conditions. Sometimes, the cell cannot survive to this process, and autophagic cell death is activated (Fulda, 2012). But in other times, the aim of the autophagy, which is to survive in extreme conditions, is performed (Hu et al., 2012). Contrary to the K-562 cell line, in lymphoblastoid cells, although autophagy is activated, cell death is not induced while a decrease in cell proliferation rate is observed. One possibility could be the activation of the autophagic survival mechanism through the cell growth regulator mTOR protein, the same as it was described after nutrient deprivation in different type of cells (Jung et al., 2010). A decrease in cell proliferation by autophagic activation was also observed in colon cells and in the human mammary epithelial cell line after treatment with rapamycin and hydrogen sulfide, respectively (Wu et al., 2012; Chen et al., 2013). Also, rapamycin has shown cell cycle arrest in G1 phase of the mitosis in endometrial carcinomas (Bae-Jump et al., 2010). Therefore, the activation of autophagy in the lymphoblastoid cell line leads to a proliferation rate arrest due to the YTX treatment. This theory is corroborated by the results shown in Figure 8, since K-562 cells number is decreased by YTX effect, while lymphoblastoid cells number is not modified after YTX incubation, suggesting cellular proliferation arrest by the toxin.

It was described that the point where cell proliferation, apoptosis, and autophagy converge are the mitochondria (Filippi-Chiela et al., 2011). Therefore, a close relationship between MPTP and autophagy or apoptosis was established (Lemasters et al., 1998). MPTP leads to mitochondrial membrane depolarization that finally induces the opening transition pore to release pro-apoptotic proteins to the cytosol (Weiss et al., 2003). When the MPTP process is activated only in some mitochondria, the autophagy is triggered, while apoptosis is activated when a high number of mitochondria have MPTP activation (Lemasters et al., 1998). But the exact linking mechanism between MPTP and autophagy is unknown (Elmore et al., 2001). In his work, after 48 h YTX treatment, when autophagic cell death is activated in the K-562 line, mitochondrial mass was decreased. In this context, mitophagy was defined as the mitochondrial autophagy to describe the mitochondrial removal, characteristic of autophagy activation (Youle and Narendra, 2011). On the other hand, after the incubation with YTX for 48 h, the mitochondrial mass was increased in the lymphoblastoid cell line. Therefore, this is another hallmark to guarantee that the autophagic process activated after 48 h in the K-562 cell line is not occurring in the lymphoblastoid cell line. In the first case, the cells activate autophagy accompanied by mitophagy and mitochondrial signal decrease, while the lymphoblastoid cells could activate autophagy as a survival process accompanied by an increase of mitochondrial mass.

In conclusion, YTX modulates different pathways with different final result depending on the cellular model. In the tumor model, K-562 cells, the type II of programmed cell death is triggered after YTX treatment. While in a non-tumor model, the lymphoblastoid cell line, a survival autophagic process is activated after toxin incubation. Furthermore, apoptosis is only triggered in the tumor model, while in lymphoblastoid cell line, this type of cell death was not activated after YTX incubation. This fact could be interesting in order to study the potential effect of YTX in different anti-tumor therapeutics treatments. All these results are summarized in Table 1.

**Table 1:** **Summary of the viability, apoptotic and autophagic features of K-562 and lymphoblastoid cell line after YTX incubation studied in this paper**.

**Features**	**K-562 tumor cell line**		**Lymphoblastoid cell line**	
	**24 h**	**48 h**	**24 h**	**48 h**
MTT viability	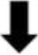	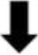	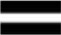	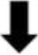
LDH release	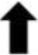	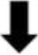	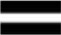	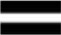
PDE4A expression	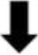	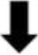	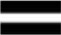	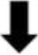
Mitochondrial mass	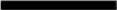	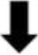	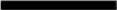	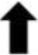
Cell death	YES	YES	NO	NO
Apoptotic hallmarks	YES	NO	NO	NO
Autophagic hallmarks	NO	YES	NO	YES
Number of cells	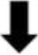	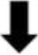	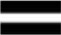	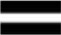
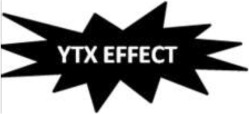	Apoptosis cell death	Autophagic cell death	No effect	Cell proliferation arrested by autophagy cell survive

### Conflict of interest statement

The authors declare that the research was conducted in the absence of any commercial or financial relationships that could be construed as a potential conflict of interest.
